# *Lactiplantibacillus plantarum* Lp05 ensures immune developmental safety and protects against colitis via promoting Treg differentiation in mice

**DOI:** 10.1128/spectrum.00043-26

**Published:** 2026-09-28

**Authors:** Shengning Liang, Yanjie Chen, Yixiang Zhang, Yuxin Zhang, Zehua He, Jia Dong, Xu Cao, Yongquan Li, Wencan Zhang, Jianjun Hu

**Affiliations:** 1 Shanghai Key Laboratory of Veterinary Biotechnology, School of Agriculture and Biology, Shanghai Jiao Tong University 117750, Shanghai, China; 2 Department of Infectious Disease, Tongren Hospital, Shanghai Jiao Tong University School of Medicine 56694, Shanghai, China; 3 Shanghai Frontiers Science Center for Drug Target Identification and Delivery, and the Engineering Research Center of Cell and Therapeutic Antibody of the Ministry of Education, School of Pharmaceutical Sciences, Shanghai Jiao Tong University 12474, Shanghai, China; 4 College of Horticulture and Landscape Architecture, Zhongkai University of Agriculture and Engineering 47894, Guangzhou, China; 5 Department of Infectious Diseases, Shanghai Pudong Hospital, Fudan University Pudong Medical Center 542170, Shanghai, China; LSU Health Shreveport, Shreveport, Louisiana, USA

**Keywords:** Lp05, adaptive immunity, T cell development and maturation, inflammation, colitis

## Abstract

*Lactiplantibacillus plantarum* (formerly *Lactobacillus plantarum*) Lp05, a strain isolated from pickled vegetables, is widely used as a probiotic in the food industry. However, its impact on the host immune system, especially the adaptive immune system, remains poorly understood. In this study, we systematically evaluated the influence of Lp05 on T cell immunity under both homeostatic and inflammatory conditions in mice. First, we showed that early life exposure to Lp05 does not compromise the development or maturation of the adaptive immune system, as evidenced by normal thymic T cell development and unaltered immune cell profiles in the spleen and lymph nodes of offspring from Lp05-treated dams. Second, in an inflammatory setting of an inflammatory bowel disease model, Lp05 supplementation protected mice against dextran sulfate sodium-induced colitis. Mechanistically, this protection was correlated with an increased differentiation of CD4^+^Foxp3^+^ Treg cells, likely associated with enhancement of microbial amino acid biosynthesis and metabolic pathways in the gut. Taken together, our findings demonstrate that Lp05 administration is safe for T cell development and exhibits therapeutic potential in colitis, primarily by promoting Treg cell differentiation.

**IMPORTANCE:**

Despite the growing use of probiotics in immune-targeted products, their specific effects on adaptive immunity are not well defined. This study systematically evaluates the probiotic strain *Lactiplantibacillus plantarum* Lp05, examining its influence on T cell immunity during both early-life development and inflammatory disease. We show that perinatal Lp05 exposure does not adversely affect immune development. In a model of inflammatory bowel disease, Lp05 supplementation effectively attenuated dextran sulfate sodium-induced colitis. This protection was associated with a marked increase in intestinal Treg cell differentiation, potentially mediated through enhanced microbial amino acid biosynthesis. Importantly, these findings confirm that a food-origin probiotic can be safely administered during immune development while exerting therapeutic benefits via Treg induction, bridging key gaps between probiotic safety, immune modulation, and clinical potential.

## INTRODUCTION

As a vital component of host immunity, the adaptive immune system provides targeted and long-lasting protection. By generating highly specific antibodies and cell-mediated attacks, primarily through B and T lymphocytes, it allows the body to mount a tailored defense against a vast array of bacteria, viruses, and other threats, and ensures long-term protection (1, 2). Within the adaptive immune system, T lymphocytes (T cells) constitute central players, orchestrating cell-mediated immune responses. The fine-tuned development, maturation, and differentiation of T cell subsets are critical for immune homeostasis (3). Dysregulation of the balance between proinflammatory T cell subsets (T helper 1 [Th1], Th2, and Th17) and T regulatory cells (Treg) serves as a central mechanism in many autoimmune diseases. For instance, inflammatory bowel disease (IBD), a chronic relapsing gastrointestinal disorder characterized by persistent inflammation in the gastrointestinal tract, is driven by an expansion of pro-inflammatory Th1/Th17 cells coupled with a deficit in suppressive Tregs (4, 5).

It is known that the gut microbiota plays a fundamental and multifaceted role in shaping the development, differentiation, and function of the host’s immune system. Previous research has revealed that antibiotic-treated and germ-free mice express distinct intestinal T cell receptor repertoires compared to mice with a normal microbiota composition, suggesting that microbial antigens alter T cell development (6, 7). In the newborn, the microbiome and immune system develop in parallel and influence each other. Alterations of the microbiome can lead to far-reaching changes in the development of the immune system and thus preprogram the newborn’s susceptibility to later inflammatory diseases (8, 9). Studies indicate that the colonization of the infant gut microbiota is initiated primarily by the vertical transmission of maternal gut microbes (10). Mother-to-infant gut microbiome transmission is significant and stable during infancy, which serves as the core pathway for infant gut microbiota colonization, particularly dominating the early life stages (11).

Probiotics are defined as live microorganisms which, when administered in adequate amounts, confer a health benefit to the host. They have beneficial effects on a wide range of diseases such as IBD, allergies, obesity, autoimmune diseases, and many others (12). *Lactiplantibacillus plantarum* is well studied as a probiotic, having a long history of safe use in various foods. *Lactiplantibacillus plantarum* Lp05 (hereafter referred to as “Lp05”), a strain isolated from pickled vegetables, has been demonstrated to protect against ethanol-induced liver injury in zebrafish through metabolic and microbiota modulation as well as to enhance the host defense against *Helicobacter pylori* infection through modulation of the gastrointestinal microenvironment (13, 14). However, its impact on the host immune system, especially the adaptive immune system, has yet to be explored.

This study systematically evaluates the influence of Lp05 on adaptive immunity in mice under both homeostatic and inflammatory conditions. Specifically, we investigate its effects on early life immune development and its potential to modulate T cell differentiation using an IBD model. Ultimately, this work seeks to elucidate the probiotic mechanisms of Lp05, with a particular focus on its regulation of T cell immunity. The findings may provide mechanistic insights into how specific *Lactiplantibacillus* strains shape host adaptive immune responses and support their rational application in immune-related disorders.

## MATERIALS AND METHODS

### Probiotic strain and culture

The probiotic strain *Lactiplantibacillus plantarum* Lp05 (Lp05) used in this study was isolated from pickled vegetables. It was provided by Microhealthcare (Suzhou) Co., Ltd. (Suzhou, China) in the form of lyophilized powder, with an activity of 90 billion CFU/g. Prior to the experiment, the bacteria were activated by culturing 100 mg of the powder in 20 mL of MRS broth under static conditions at 37°C for 26 h. Cells were harvested by centrifugation (8,000 rpm, 10°C, 20 min), washed, and resuspended in 2 mL of fresh MRS broth. The suspension was mixed with an equal volume of 50% glycerol (final concentration of glycerol is 25%) and stored at −80°C in cryovials as working stocks. Lp05 was cultivated by dissolving 20 μL frozen stock in 20 mL fresh MRS broth statically at 37°C. Sampling was conducted every 2 h for the determination of viable bacterial counts by measuring the OD₆₀₀ of the culture with SpectraMax iD3/iD5 microplate reader (Molecular Devices). Bacteria were harvested for oral gavage at the stationary phase.

### Mice

Healthy C57BL/6J mice were used in this study. All animals were obtained from Jiangsu GemPharmatech Co., Ltd. (Nanjing, China) and maintained under specific pathogen-free conditions at the Animal Resource Center of Shanghai Jiao Tong University. All procedures were approved by the university’s Institutional Animal Care and Use Committee (IACUC# A2023233-005). Prior to any experimental manipulation, the mice underwent a 7-day acclimation period. When necessary, anesthesia was induced by exposing the animals to 3% isoflurane inside an induction chamber (RWD AIJI-IE, Shenzhen, China), ensuring a painless and stress-free process. At the end of the experiment, mice were humanely euthanized via CO_2_ inhalation, followed by cervical dislocation to confirm death. This protocol was approved by the IACUC of Shanghai Jiao Tong University and complies with American Veterinary Medical Association guidelines.

### Antibodies

Antibodies for flow cytometry, including Brilliant Violet (BV) 605-conjugated anti-CD4 (dilution ratio, 1:100; RM4-5) and BV 421-conjugated anti-CD3 (dilution ratio, 1:100; 145-2C11), were from BioLegend. Allophycocyanin (APC)-conjugated anti-CD19 (dilution ratio, 1:100; 6D5) and fluorescein isothiocyanate-conjugated anti-CD45 (dilution ratio, 1:100; 30F11) were from Miltenyi Biotec. APC-conjugated anti-Foxp3 (dilution ratio, 1:100; FJK-16s) was from Invitrogen.

### Flow cytometry

Tissues of spleen, lymph nodes, and thymus were aseptically collected from mice and mechanically dissociated through a 70 μm strainer. Splenocytes were treated with RBC lysis buffer for 3 min at room temperature. All cells were washed, filtered, and resuspended in PBS for analysis.

The cell staining protocol for flow cytometry was adapted from our previously published method (15). Specifically, for surface staining, cells were directly stained with antibodies in PBS at 4°C for 15 min. For transcription factor Foxp3 staining, cells pre-stained with surface markers were fixed and permeabilized in TF Fix/Perm buffer (BD Biosciences) at 4°C for 20 min, washed once with TF Perm/Wash buffer, and stained with target markers in the TF Perm/Wash buffer at 4°C for 15 min. The expression of surface and intracellular markers was analyzed with a BD LSR Fortessa flow cytometer.

### Oral administration of Lp05 to dams

C57BL/6J mice of 6–8 weeks were mated. Starting at embryonic day 9.5, dams were assigned to three groups: (i) the Lp05-treated group, which received a daily oral gavage of 1.5 × 10⁸ CFU of Lp05 in 200 µL PBS per mouse; (ii) the vehicle control group, receiving a daily oral gavage of 200 µL PBS only per mouse; and (iii) the untreated blank group, which underwent no gavage procedure. The dose of Lp05 was chosen based on a review of the literature and study purpose. The gavage protocol was continued daily until the pups were harvested. All mice had *ad libitum* access to standard chow and drinking water. Body weight of dams was monitored daily. Starting from 3 weeks of age, pups were weighed weekly until 6 weeks of age.

### Dextran sulfate sodium-induced colitis in mice

Dextran sulfate sodium (DSS, molecular weight 36–50 kDa) was obtained from MP Biomedicals. The mice were assigned to three groups: Lp05 treated (Lp05), DSS model (DSS), and healthy control (HC). Mice in the Lp05 group received 1 × 10⁹ CFU of Lp05 suspended in 200 μL of PBS daily by gavage for 30 consecutive days, whereas mice in the DSS group were given 200 μL of PBS over the same period. For the first 12 days, both treatment groups were provided with normal water. Starting on day 13, normal water was replaced with a DSS regimen consisting of three cycles of 2% (wt/vol) DSS for 4 days followed by normal water for 2 days. Throughout the experimental period of 30 days, the HC group received normal drinking water daily. The dose of Lp05 was chosen based on a review of the literature and supported by previous probiotic studies in colitis models. Body weight of mice was recorded each day to monitor the animals’ health and to confirm successful induction of colitis. On day 30, the mice were euthanized, and samples of spleen, mesenteric lymph nodes (mLNs), colon, and feces were collected for further analysis. Colon length was also measured as a physiological indicator.

### Histological evaluation of colon

Colon samples were initially washed with PBS, then fixed in 4% (wt/vol) paraformaldehyde (Servicebio, Wuhan, China), followed by dehydration and paraffin embedding. The specimens were sectioned into 4 μm slices and subsequently stained with hematoxylin and eosin (H&E).

### Fecal metagenomic sequencing

Fecal samples from four mice per group were selected for metagenomic sequencing. Microbial DNA was extracted using the QIAamp DNA Stool Mini Kit (QIAGEN), and DNA integrity was verified by 0.8% agarose gel electrophoresis. Sequencing was performed by BGI Group (Shenzhen, China). Library preparation was carried out with the BGI Optimal DNA Library Prep Kit: genomic DNA was fragmented, size-selected with magnetic beads, end-repaired, A-tailed, and ligated to adapters. After PCR amplification and quality control, the double-stranded library was denatured and circularized to obtain single-stranded circular DNA. Linear DNA was digested, and the circular library was amplified via rolling circle amplification with phi29 to generate DNA nanoballs (DNBs), each containing ~300 copies of the original library molecule. DNBs were loaded onto a patterned nanoarray, and 150 bp paired-end reads were generated on the DNBSEQ-T7 platform (BGI).

### Data analysis

Flow cytometry data were processed and plotted using FlowJo 10.8.1. H&E-stained colon sections were examined with SlideViewer. Other data were analyzed and graphed using GraphPad Prism 9.5. One-way analysis of variance (ANOVA) followed by Tukey’s multiple comparisons test and two-tailed Student’s *t*-test were applied as appropriate. Statistical significance is indicated as ns (*P* > 0.05), * (*P* < 0.05), ** (*P* < 0.01), and *** (*P* < 0.001).

## RESULTS

### Oral administration of Lp05 to dams has no adverse effects on both dams and pups

**Fig 1:**
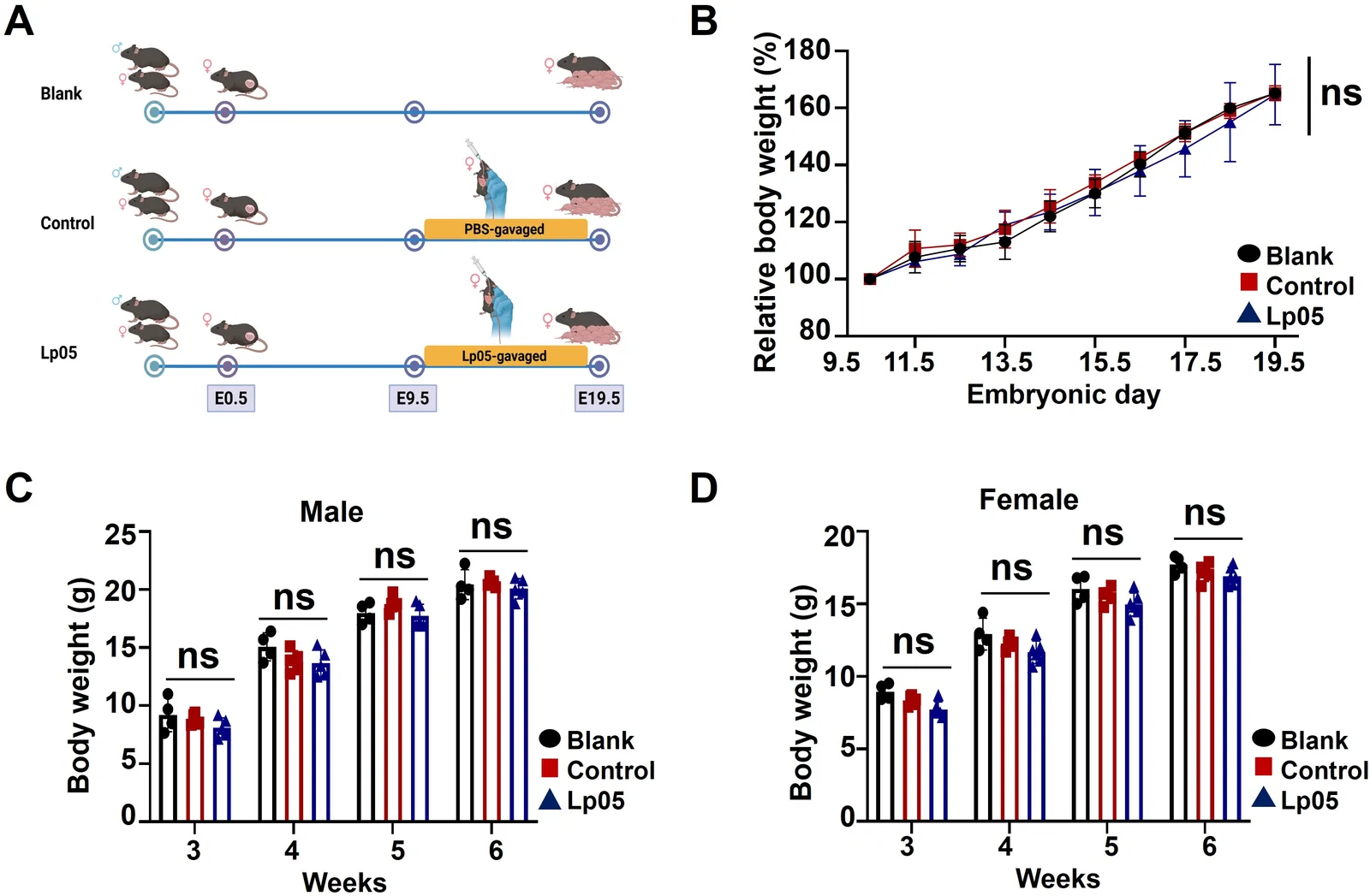
Oral administration of Lp05 to dams has no adverse effects on both dams and pups. (**A**) Schematic overview of the experimental gavage procedure used during pregnancy. (**B**) Relative body weight of Blank dams and dams subject to PBS gavage and Lp05 gavage from embryonic day 9.5 (E9.5) until delivery (*n* ≥ 4 per group). (**C**) Body weight of male offspring born to Blank dams, Control dams, and Lp05-treated dams at different ages (*n* ≥ 4 per group). (**D**) Body weight of female offspring born to Blank dams, Control dams, and Lp05-treated dams at different ages (*n* ≥ 4 per group). Data are presented as mean ± SD. ns, not significant (one-way ANOVA followed by Tukey’s multiple-comparisons test).

Daily oral administration of Lp05 (1.5 × 10^8^ CFU per day in 200 μL PBS) was performed from embryonic day 9.5 (E9.5) until delivery, as outlined in Fig. 1A. The Blank group received no intervention, while the Control group received an equal volume of PBS following the same schedule. As shown in Fig. 1B, no significant difference in body weight gain was observed among the Blank, Control, and Lp05-treated groups during gestation, indicating no adverse effect of Lp05 on maternal health. After weaning (at 3 weeks of age), pup body weights were monitored weekly until 6 weeks of age. As presented in Fig. 1C (males) and Fig. 1D (females), there was no significant difference in growth rate across the three groups at any time point (one-way ANOVA, *P* > 0.05). These data collectively demonstrate that prenatal administration of Lp05 had no detectable adverse effects on either dams or pups under the conditions tested.

### Oral administration of Lp05 to dams did not affect the development or maturation of immune cells in the offspring

**Fig 2:**
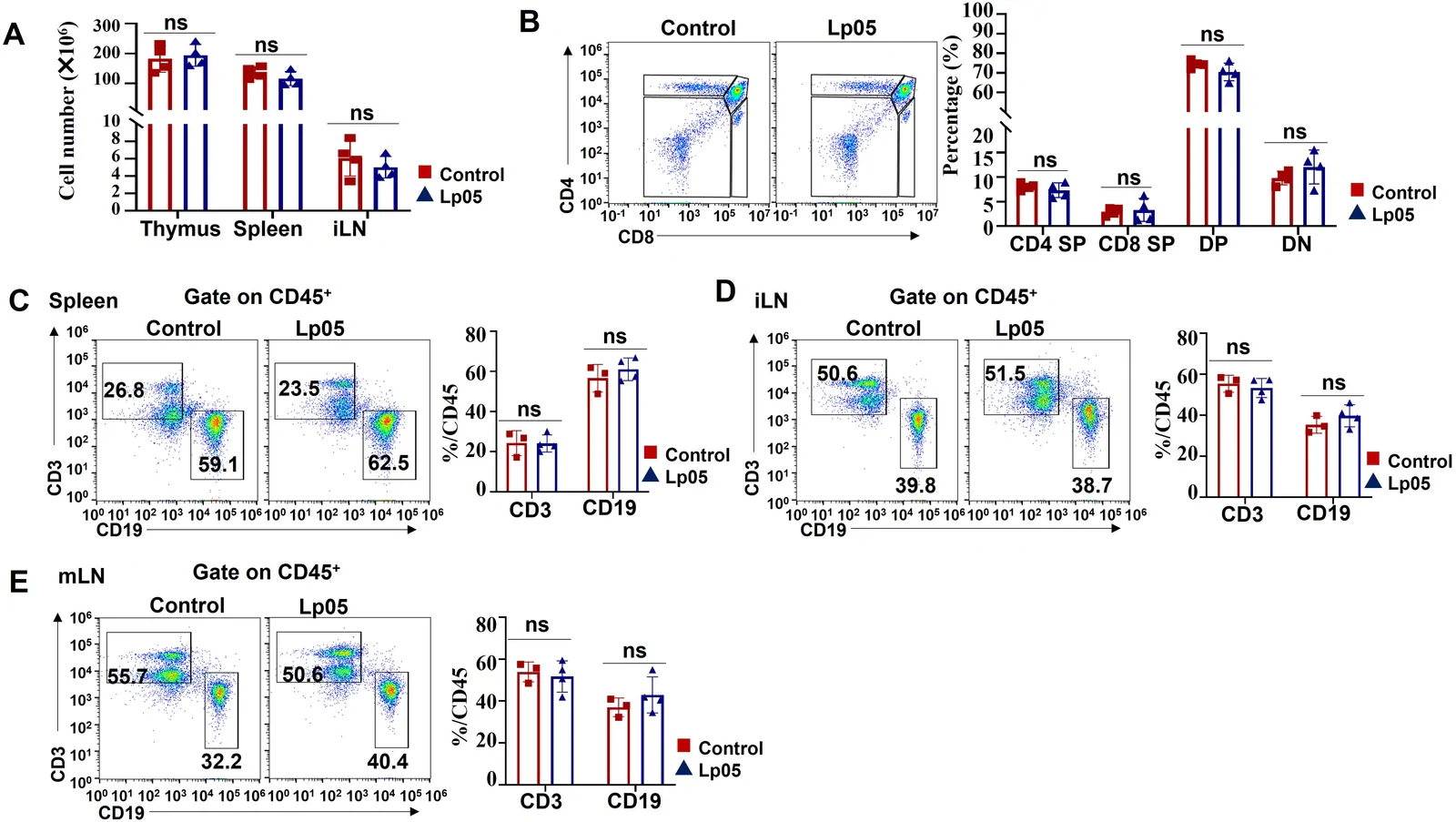
Oral administration of Lp05 to dams did not affect the development or maturation of immune cells in the offspring. (**A**) Total cell number from thymus, spleen, and iLN of offspring born to Control dams and Lp05-treated dams (*n* ≥ 4 per group). (**B**) Analysis of CD4^−^CD8^−^ double negative (DN), CD4^+^CD8^+^ double positive (DP), CD4 or CD8 single positive (SP) thymocytes from offspring born to indicated dams (*n* ≥ 4 per group); the left panel shows representative flow cytometry plots, and the right panel shows the quantified percentages of the indicated population from all mice. (**C**) The distribution of CD3^+^ T cells and CD19^+^ B cells among CD45^+^ cells in spleen from offspring born to indicated dams (*n* ≥ 3 per group); the left panel shows representative flow cytometry plots, and the right panel shows the quantified percentages of the indicated population from all mice. (**D**) The distribution of CD3^+^ T cells and CD19^+^ B cells among CD45^+^ cells in iLN from offspring born to indicated dams (*n* ≥ 3 per group); the left panel shows representative flow cytometry plots, and the right panel shows the quantified percentages of the indicated population from all mice. (**E**) The distribution of CD3^+^ T cells and CD19^+^ B cells among CD45^+^ cells in mLN from offspring born to indicated dams (*n* ≥ 3 per group); the left panel shows representative flow cytometry plots, and the right panel shows the quantified percentages of the indicated population from all mice. Boxed area: cell population of interest. Data are presented as mean ± SD. ns, not significant (two-tailed Student’s *t*-test).

Major immune organs, including thymus, spleen, inguinal lymph nodes (iLN), and mLNs, were collected from pups at 6–7 weeks of age for analysis. As shown in Fig. 2A, the total cell numbers in the thymus, spleen, and iLN were comparable between offspring from the Control and Lp05-treated groups, suggesting that prenatal exposure to Lp05 does not alter fundamental immune organ development.

We next analyzed T cell subpopulations in the thymus from offspring, and the data are shown in Fig. 2B. The thymus is the primary lymphoid organ where bone marrow-derived precursor cells develop into mature, self-tolerant, and functionally competent T lymphocytes. T cell development proceeds sequentially from CD4^−^CD8^−^ double-negative (DN) progenitors to CD4^+^CD8^+^ double-positive (DP) cells, which undergo selection and mature into single positive (SP; CD4^+^ or CD8^+^) T cells before emigrating to the periphery (16). We observed no significant differences between the Control and Lp05-treated groups in the distribution of DN, DP, and SP thymocyte subsets, both of which were consistent with normal thymic development, indicating that Lp05 did not disrupt this tightly regulated T cell maturation process.

Furthermore, pups born to Lp05-treated dams exhibited normal cellular profiles in the spleen, iLNs, and mLNs. The relative abundances of CD19^+^ B cells and CD3^+^ T cells in these tissues remained within normal ranges compared to controls. These results suggest that oral administration of Lp05 to pregnant dams does not perturb the fundamental development or maturation of adaptive immune cells in their offspring.

### Lp05 administration provided protection to mice in DSS-induced colitis model

**Fig 3:**
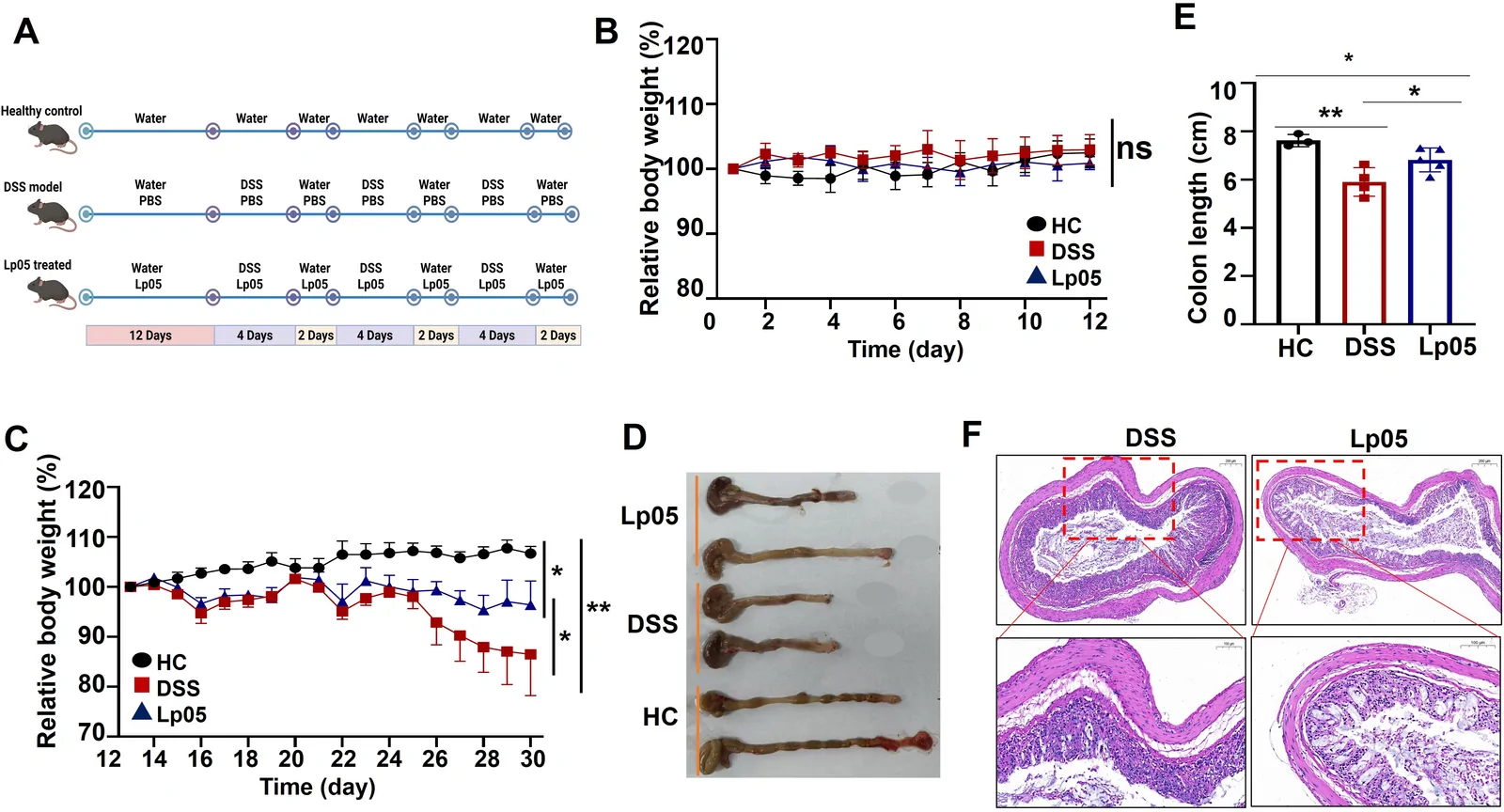
Lp05 administration provided protection to mice in the DSS-induced colitis model. (**A**) Overview of the experimental settings used in the DSS-induced colitis model. (**B**) Body weight of mice from healthy control (HC) group, DSS model (DSS) group, and Lp05 treated (Lp05) group in the first 12 days (*n* ≥ 3 per group). (**C**) Body weight of mice from HC group, DSS group, and Lp05 group from days 12 to 30 (*n* ≥ 3 per group). (**D**) Representative image of colons from HC group, DSS group, and Lp05 group. (**E**) Colon length (cm) from HC group, DSS group, and Lp05 group (*n* ≥ 3 per group). (**F**) Section of hematoxylin and eosin-stained colon from Control group, DSS group, and Lp05 group. Data in panel C are presented as means ± SEM, while data in panels B and E are presented as means ± SD. *P < 0.05; **P < 0.01; ns, not significant (two-tailed Student’s *t*-test).

Figure 3A illustrates the protocol used to establish the DSS-induced colitis model. We found that Lp05 was well tolerated by the mice during the adaptation period of gavage administration, as after 12 days of continuous oral administration, no significant differences from the HC group were detected in body weight (Fig. 3B).

As shown in Fig. 3C, mice in the DSS group experienced a progressive drop in body weight beginning on day 16, with values falling significantly below those of the HC group, evidence that colitis was successfully induced. Crucially, relative to the DSS group, Lp05-treated mice showed substantially less weight loss (Fig. 3C), showing that Lp05 administration conferred significant protection to mice within the DSS-induced colitis model.

Colon shortening serves as a reliable physical metric of colitis intensity, with greater shortening reflecting more extensive inflammatory destruction. As anticipated, colon length in the DSS group was significantly reduced compared with the HC group (Fig. 3D and E). Remarkably, Lp05 administration prevented this shortening to a significant degree relative to the DSS group (Fig. 3D and E), highlighting its protective capacity.

Consistent with the above observed phenotypes, histological analysis (Fig. 3F) of H&E-stained colonic sections revealed severe tissue damage in the DSS group. This includes mucosal erosion, heavy infiltration of inflammatory cells, loss of normal architecture, and a thickened mucosal wall, all of which are classic signs of advanced colitis. By contrast, Lp05 treatment markedly diminished these histopathological abnormalities, clearly limiting inflammatory injury. Collectively, these results strongly indicate that Lp05 provides strong protection against colon damage associated with colitis.

### Lp05 administration upregulated the differentiation of CD4^+^Foxp3^+^ Treg cells in the DSS-induced colitis model

**Fig 4:**
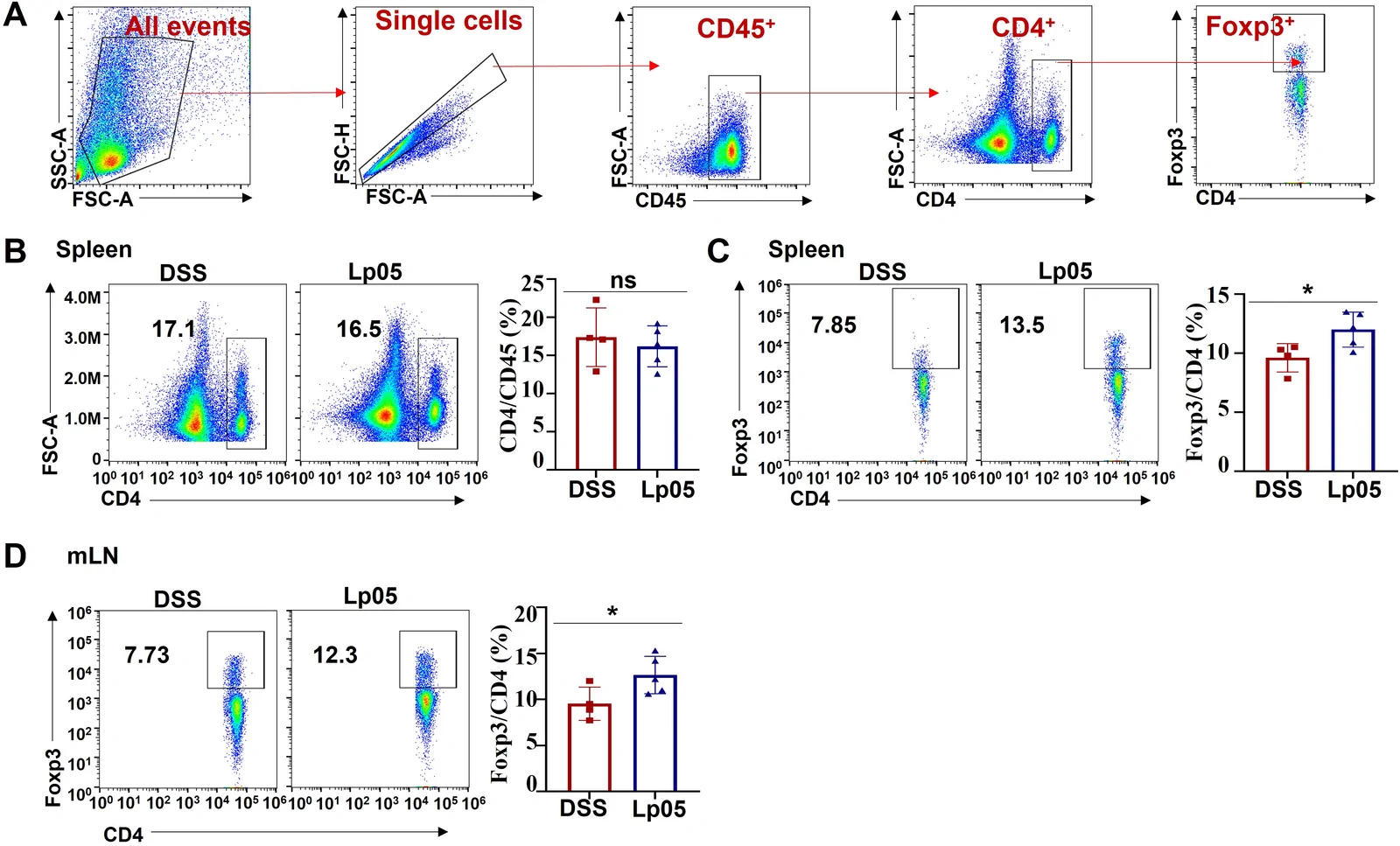
Lp05 administration upregulated the differentiation of CD4^+^Foxp3^+^ Treg cells in the DSS-induced colitis model. (**A**) Gating strategy for Fig. **4B–D**. (**B**) CD4^+^ cells among CD45^+^ cells in the mice spleen from DSS group and Lp05 group (*n* ≥ 4 per group); the left panel shows representative flow cytometry plots, and the right panel shows the quantified percentages of the indicated population from all mice. (**C**) Foxp3^+^ cells among CD4^+^ cells in the mice spleen from DSS group and Lp05 group (*n* ≥ 4 per group); the left panel shows representative flow cytometry plots, and the right panel shows the quantified percentages of the indicated population from all mice. (**D**) Foxp3^+^ cells among CD4^+^ cells in the mice mesenteric lymph nodes (mLNs) from DSS group and Lp05 group (*n* ≥ 4 per group); the left panel shows representative flow cytometry plots, and the right panel shows the quantified percentages of the indicated population from all mice. Boxed area: cell population of interest. Data are presented as means ± SD. *P < 0.05; **P < 0.01; ns, not significant (two-tailed Student’s *t*-test).

CD4^+^Foxp3^+^ Treg cells are immunosuppressive and inhibit proinflammatory activity through various mechanisms, including the secretion of IL-10 and TGF-β, as well as direct cell-contact suppression (16). The dysfunction of Treg subsets is a recognized key factor driving IBD (17, 18). Thus, flow cytometry was subsequently applied to examine immune cell profiles across the different treatment groups, with a specific focus on CD4^+^Foxp3^+^ Treg cells. The gating strategy is shown in Fig. 4A. Briefly, cells were sequentially gated on CD45^+^ and CD4^+^ populations, and Foxp3 expression was analyzed within the CD4^+^ gate.

According to Fig. 4B, there was no significant difference in the proportion of total splenic CD4^+^ T cells between the DSS control group and the Lp05-treated group, suggesting that Lp05 did not lead to a broad increase in CD4^+^ T cell numbers. However, upon Lp05 treatment, Foxp3 expression within the CD4^+^ T cell population rose notably, both in the spleen (Fig. 4C) and in the mLNs (Fig. 4D). Taken together, these findings imply that the protective effects observed in the DSS-induced colitis model are coincided with Lp05-promoted Treg expansion, suggesting a potential contribution, which may subsequently reduce the proinflammatory signals.

### Lp05 administration modified microbial metabolic pathways in the DSS-induced colitis model

**Fig 5:**
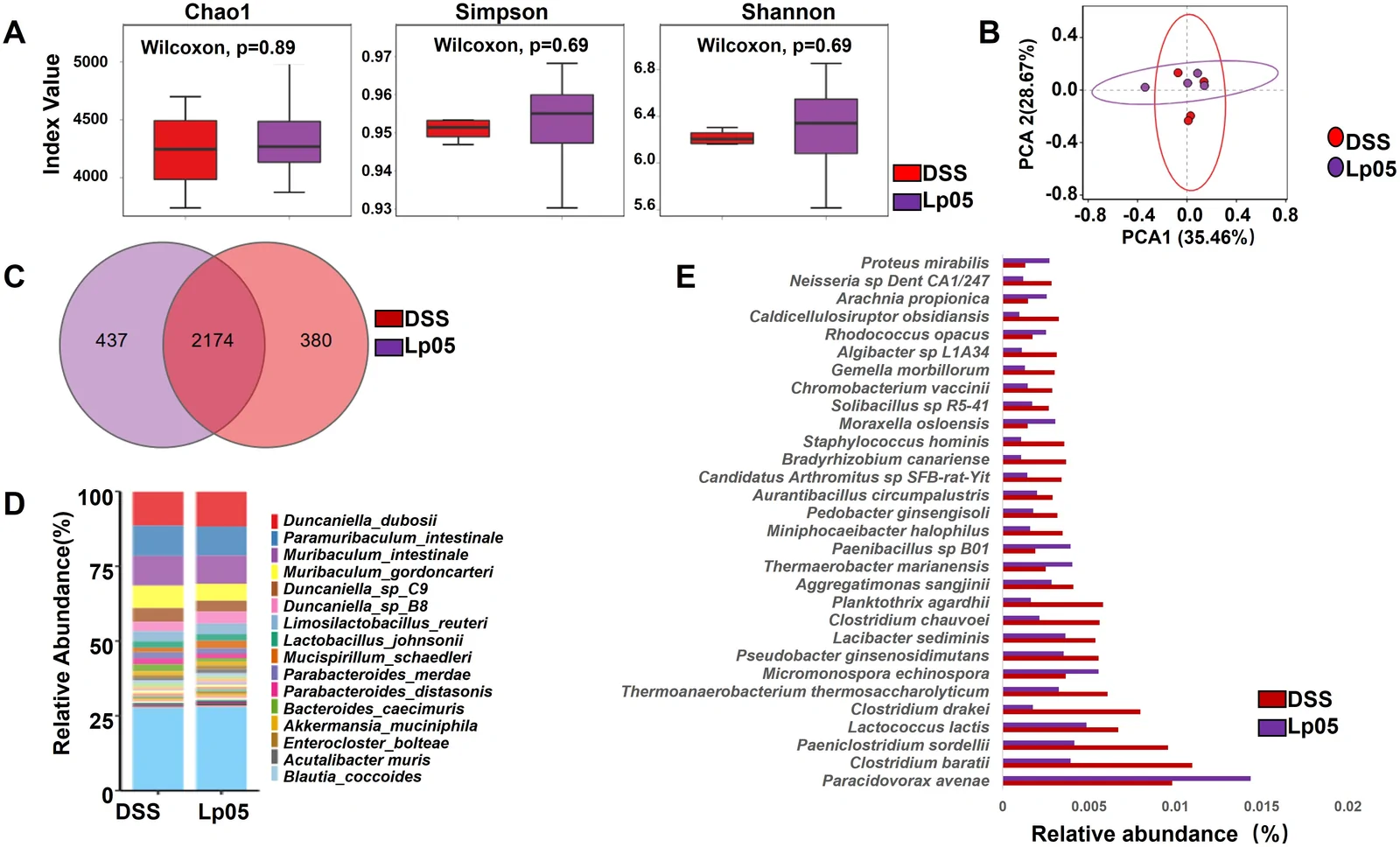
Lp05 administration has a minimal impact on the overall colonic microbiota structure. (**A**) Box plots of α-diversity in colon microbiota at the species level from DSS group and Lp05 group. (**B**) The PCoA plots of β-diversity between rectum microbiota from DSS group and Lp05 group using Bray-Curtis distance. (**C**) Comparison of the total species in gut microbiota from DSS group and Lp05 group. (**D**) Bar plot of species-level relative frequency in gut microbiota from DSS group and Lp05 group. The top 16 most abundant species were shown. (**E**) Relative abundance of the differentially distributed gut microbiota from DSS group and Lp05 group. The top 30 most abundant species were shown.

As gut microbiota has been well demonstrated to play a critical role in colitis (6, 19), we next performed metagenomic sequencing to assess whether the protective action of Lp05 in the DSS-induced colitis model was linked to changes in the colonic microbial composition. At the species level, no significant differences in α-diversity were observed between the DSS control and Lp05-treated samples, whether measured by the Chao1, Simpson, or Shannon index (Fig. 5A). Furthermore, β-diversity analysis using Principal Coordinates Analysis (PCoA) failed to separate samples by treatment group, pointing to a lack of substantial differences in species abundance (Fig. 5B). Taken together, these findings argue that the probiotic Lp05 only modestly affects the overall composition of the colonic microbiota.

A total of 2,991 species were identified across all samples, with 2,174 species shared between the DSS model and Lp05-treated groups (Fig. 5C). Furthermore, the most prevalent species (*Duncaniella dubosii*, *Paramuribaculum intestinale*, *Muribaculum intestinale*, *Muribaculum gordoncarteri*, *Duncaniella* sp.C9, and *Duncaniella* sp.B8) were similar in both groups, as illustrated in Fig. 5D, further confirming that Lp05 only modestly affects the colonic microbial composition. However, significant differences were observed in the composition of less predominant species (Fig. 5E).

**Fig 6:**
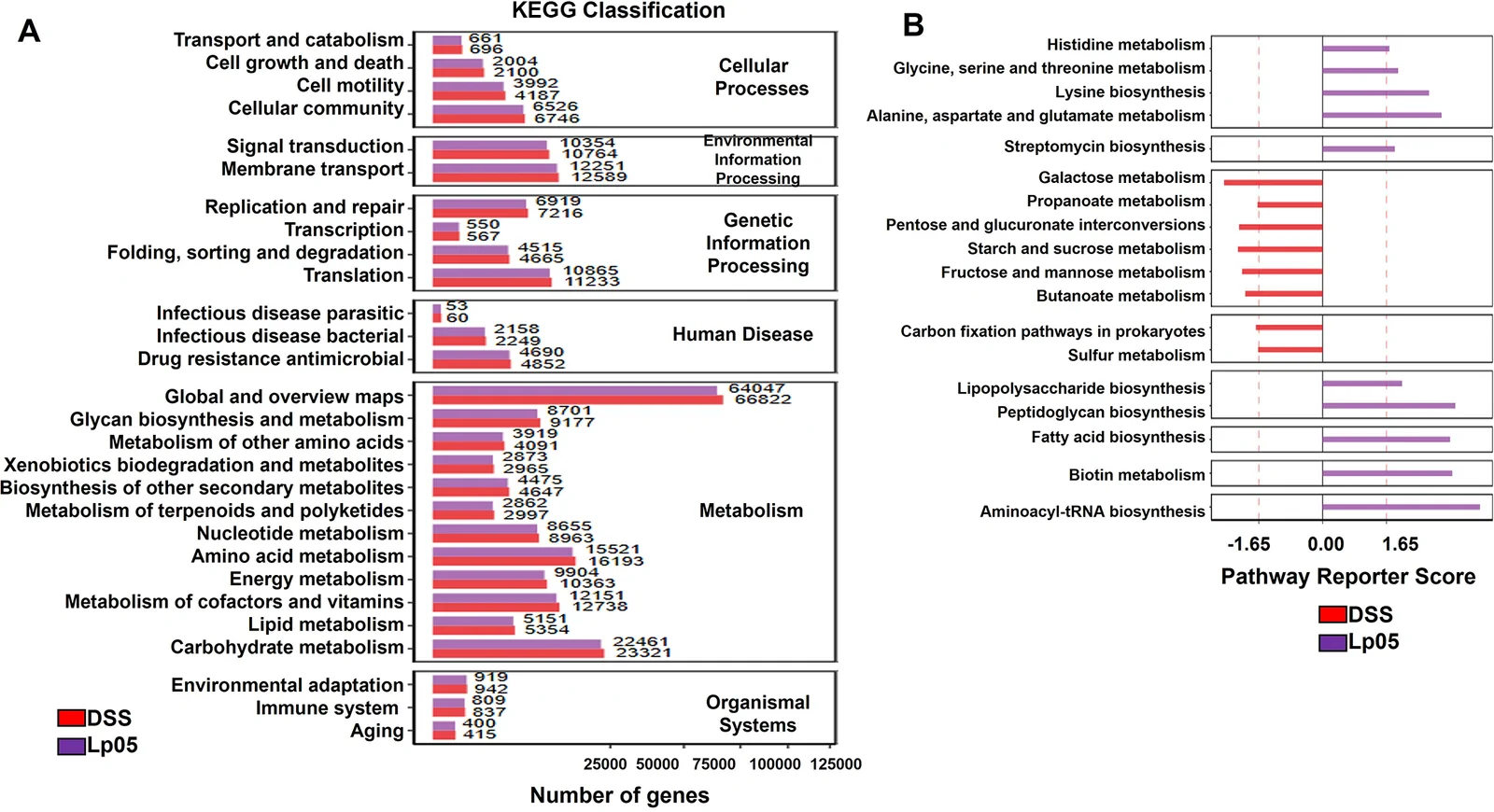
Lp05 administration modified microbial metabolic pathways. (**A**) Microbial gene enrichment based on KEGG classification. (**B**) The relative frequencies for significantly altered microbial metabolic pathways in gut microbiota from DSS group and Lp05 group. Pathways with a minimum absolute value of log_2_ fold change (log_2_FC) of 1.65 were included in the plot.

At the microbial gene level, we noticed that genes involved in metabolism were most identified (Fig. 6A). We further analyzed the microbial metabolic pathways and identified 10 pathways significantly enriched in abundance in the Lp05-treated gut microbiome, which are histidine metabolism, glycine, serine and threonine metabolism, lysine biosynthesis, alanine, aspartate, and glutamate metabolism, streptomycin biosynthesis, lipopolysaccharide (LPS) biosynthesis, peptidoglycan biosynthesis, fatty acid biosynthesis, biotin metabolism, and aminoacyl-tRNA biosynthesis, as shown in Fig. 6B. These findings indicate that Lp05 may primarily modulate the functional capacity of the gut microbiota rather than inducing a wholesale restructuring of the community.

Among those microbiome-enriched pathways, a striking observation was the enrichment of amino acid biosynthesis and metabolic pathways, implying that Lp05 boosts the gut microbiota’s biosynthetic and metabolic functions in this regard. Amino acid homeostasis is essential, and its dysregulation is closely associated with disease progression in type 2 diabetes or IBD (20, 21). They serve as both metabolic substrates and signaling molecules that critically shape T cell differentiation and function. Previous studies found that key amino acids like glutamine and leucine can promote the differentiation of proinflammatory T cell subsets such as Th1, Th2, and Th17 cells, while tryptophan catabolism fostered the development and function of Treg cells via distinct pathways (22–25). Based on this, our current finding suggests a potential hypothesis that Lp05 may modulate Treg differentiation via microbial amino acid metabolic modulation, although further metabolomic and functional studies are required to establish causation.

## DISCUSSION

Gut microbiota modulation has emerged as a promising therapeutic strategy for a range of diseases extending beyond gastrointestinal inflammatory conditions, with probiotic supplementation representing a central intervention (19, 26, 27). However, a critical gap persists in our understanding of whether and how probiotics influence adaptive immunity in immune homeostasis and inflammatory conditions. Although *Lactiplantibacillus plantarum* has a long history of safe use in various foods, and the strain of *Lactiplantibacillus plantarum* Lp05 has been demonstrated to protect against ethanol-induced liver injury in zebrafish and to enhance the host defense against *Helicobacter pylori* infection (13, 14), its impact on regulating the host immune system is yet to be explored.

Immune system development is critically shaped during early life. In this study, we first investigated the impact of very early exposure to the probiotic Lp05 on the development and maturation of the adaptive immune system. This was done by administering Lp05 to pregnant dams, anticipating vertical microbial transfer to the offspring. Throughout the monitoring period, no changes in body weight gain were observed for either dams or pups. When offspring reached 6–7 weeks of age, a stage at which the immune system is well developed, we found that pups from Lp05-treated dams exhibited normal T cell development in the thymus and normal subset composition of T cell and B cell in the major peripheral lymphoid organs. These data indicate that maternal Lp05 supplementation has no obvious impact on the establishment of baseline adaptive immunity under homeostatic conditions. This finding is reasonable, as the primary role of many probiotics may not be to fundamentally reprogram the developmental architecture of the immune system but rather to modulate its functional response, particularly during inflammatory challenges (28, 29). The absence of perturbation in steady-state immunity is, in fact, a favorable safety profile, suggesting that Lp05 exerts its protective effects without disrupting the essential and tightly regulated process of immune ontogeny. However, although previous rodent studies have shown that orally administered probiotics can be vertically transmitted to offspring during pregnancy and lactation, the present study lacks direct evidence of such transmission or colonization in pups. Therefore, our conclusions regarding early-life exposure remain tentative and require further investigation.

Next, we examined the function of Lp05 in regulating immune response in inflammatory challenges. IBD is a chronic, relapsing gastrointestinal disorder characterized by persistent inflammation, encompassing two primary subtypes—Crohn’s disease and ulcerative colitis (30, 31). Common clinical manifestations include diarrhea, abdominal pain, fatigue, anemia, reduced appetite, and unintended weight loss. Dysregulation of the balance between proinflammatory Th1/Th17 cells and suppressive Tregs in the gastrointestinal tract serves as a key driver of IBD pathogenesis (4, 5). In IBD patients, mucosal Treg depletion or impaired suppressive function is frequently observed, triggering uncontrolled inflammation that ultimately leads to irreversible damage to the intestinal mucosal barrier (32, 33). In the current study, supplementation of Lp05 provided protection to mice in the DSS-induced colitis model. Our data suggest that the effect may involve, at least in part, the promotion of CD4^+^Foxp3^+^ Treg differentiation, although further studies are needed to establish a causal link. While we focused on Tregs, other protective effects cannot be ruled out. For example, Lp05 might reinforce tight junctions or mucin production, reducing DSS penetration and subsequent inflammation, independent of Tregs. Furthermore, this probiotic could modulate other immune cells like dendritic cells, macrophages, or innate lymphoid cells, leading to secondary changes in Treg frequency without a direct Treg-centric mechanism.

Furthermore, we found that although overall α- and β-diversity remained unchanged, Lp05 administration modified microbial metabolic pathways. These findings align with the emerging view that probiotics can exert beneficial effects by modulating community functions without necessarily causing major alterations in overall microbial diversity or taxonomic composition. Such a pattern supports the concept of functional modulation rather than wholesale replacement by the probiotic strain.

A striking enrichment of amino acid biosynthesis and metabolic pathways after Lp05 treatment, which might be the underlying mechanism regulating the differentiation of T cells. Since amino acids serve as both metabolic substrates and signaling molecules that critically shape T cell differentiation and function, their availability directly influences the balance between proinflammatory and regulatory T cell subsets. Future work should be undertaken to analyze the specific mechanisms by which Lp05 promotes Treg differentiation via regulating amino acid metabolism in the context of colitis.

It is also noteworthy that Lp05 administration enriched pathways related to LPS and peptidoglycan biosynthesis, which are classically associated with proinflammatory responses. This seemingly paradoxical finding highlights the complexity of host-microbe interactions. One possible explanation is that the overall functional output of Lp05, rather than the presence of any single pathway, determines the net physiological effect. The concurrent enrichment of amino acid biosynthesis pathways may override potential proinflammatory signals, ultimately contributing to immune regulation. Nevertheless, this interpretation remains speculative and underscores the need for targeted metabolomic profiling to directly measure the levels of bioactive metabolites and their net impact on the host.

### Conclusions

Lp05, originally isolated from pickled vegetables, is widely used as a probiotic in the food industry. In this study, we evaluated the effect of Lp05 on adaptive immunity in immune homeostasis and inflammatory conditions in mice. First, we demonstrated that very early exposure to the probiotic Lp05 has no impact on the development and maturation of the adaptive immune system. This was supported by normal T cell development in the thymus and normal cellular profiles in the spleen, iLNs, and mLNs in pups born to Lp05-treated dams. Second, we examined the function of Lp05 in regulating immune response in inflammatory challenges and found that supplementation of Lp05 provided protection to mice in the DSS-induced colitis model. Mechanistically, Lp05 administration can significantly upregulate the differentiation of CD4^+^Foxp3^+^ Treg cells, probably through its boosting amino acid biosynthesis and metabolic pathways in the gut. Further studies should be taken to dissect the crosstalk between Lp05 and T cell differentiation, which will shed light on the underlying mechanisms of its protective effects.

## Data Availability

The raw metagenomic sequencing data from all samples have been deposited in the NCBI Sequence Read Archive under BioProject accession number PRJNA1465604.
